# Supplementary figures and images for: FtsZ filament structures in different nucleotide states reveal the mechanism of assembly dynamics
Source: PLoS Biol. 2022 Mar 21;20(3):e3001497. doi: 10.1371/journal.pbio.3001497 (PMC8936486; doi:10.1371/journal.pbio.3001497)

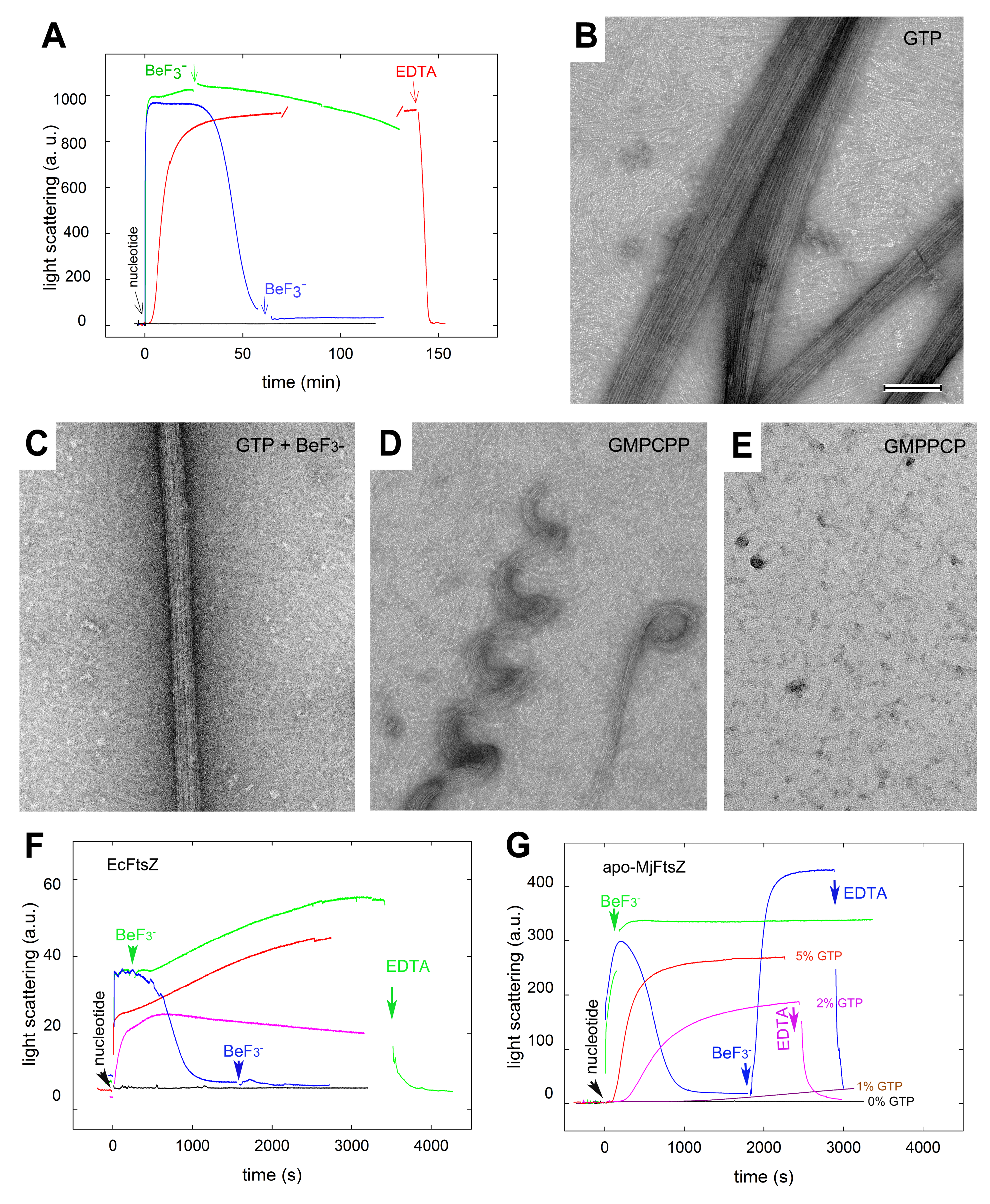

Supplement: S1 Fig — (A) Formation of polymers of SaFtsZf (49 μM), monitored by light scattering, with 1 mM GTP (blue line), with 1 mM GTP plus 5 mM BeF3− (red line), with 1 mM GTP to which 1 mM BeF3− was added at the point indicated by the arrow (green line), and with 1 mM GDP plus 5 mM BeF3− (black line). In this experiment, 10 mM MgCl2 was added to the protein samples and assembly was triggered by nucleotide addition at time 0. The sample with GTP depolymerized upon nucleotide consumption (blue line), whereas the sample containing GTP plus 5 mM BeF3− (red line) remained assembled, and disassembled with 20 mM EDTA (indicated by the red arrow). Addition of 5 mM BeF3− to unassembled SaFtsZf with GDP (black line) or following disassembly by GTP hydrolysis (blue line and arrow) was not observed to induce polymerization nucleation in these solution experiments. Numerical data for each curve can be found in S3 Data. (B) Representative electron micrograph of SaFtsZf polymers formed with GTP. (C) Polymers formed with GTP plus BeF3−. (D) Polymers formed with GMPCPP. (E) Small oligomers and protein aggregates with GMPPCP. Electron microscopy samples were collected at maximum scattering in each case. Bar: 200 nm. Experiments were made in MES assembly buffer at 25°C. (F) Light scattering traces during assembly of full-length EcFtsZ (25 μM wild-type protein) with 1 mM GTP (blue line; 5 mM BeF3− was added after depolymerization by nucleotide hydrolysis), with 1 mM GTP to which 5 mM BeF3− was added at maximal light scattering (green line, later depolymerized by 20 mM EDTA), with 1 mM GTP plus 2 mM BeF3− (pink line), with 1 mM GDP and 0.2 mM GTP plus 5 mM BeF3− (red line), and with 1 mM GDP plus 5 mM BeF3− (black line). Numerical data for each curve can be found in S4 Data. (G) Light scattering assembly time course of full-length apo-MjFtsZ (15 μM) with 1 mM GTP (blue line; 5 mM BeF3− added after depolymerization), with 1 mM GTP to which 5 mM BeF3− was added at maximal light scattering (green line), [file pbio.3001497.s001.tif]

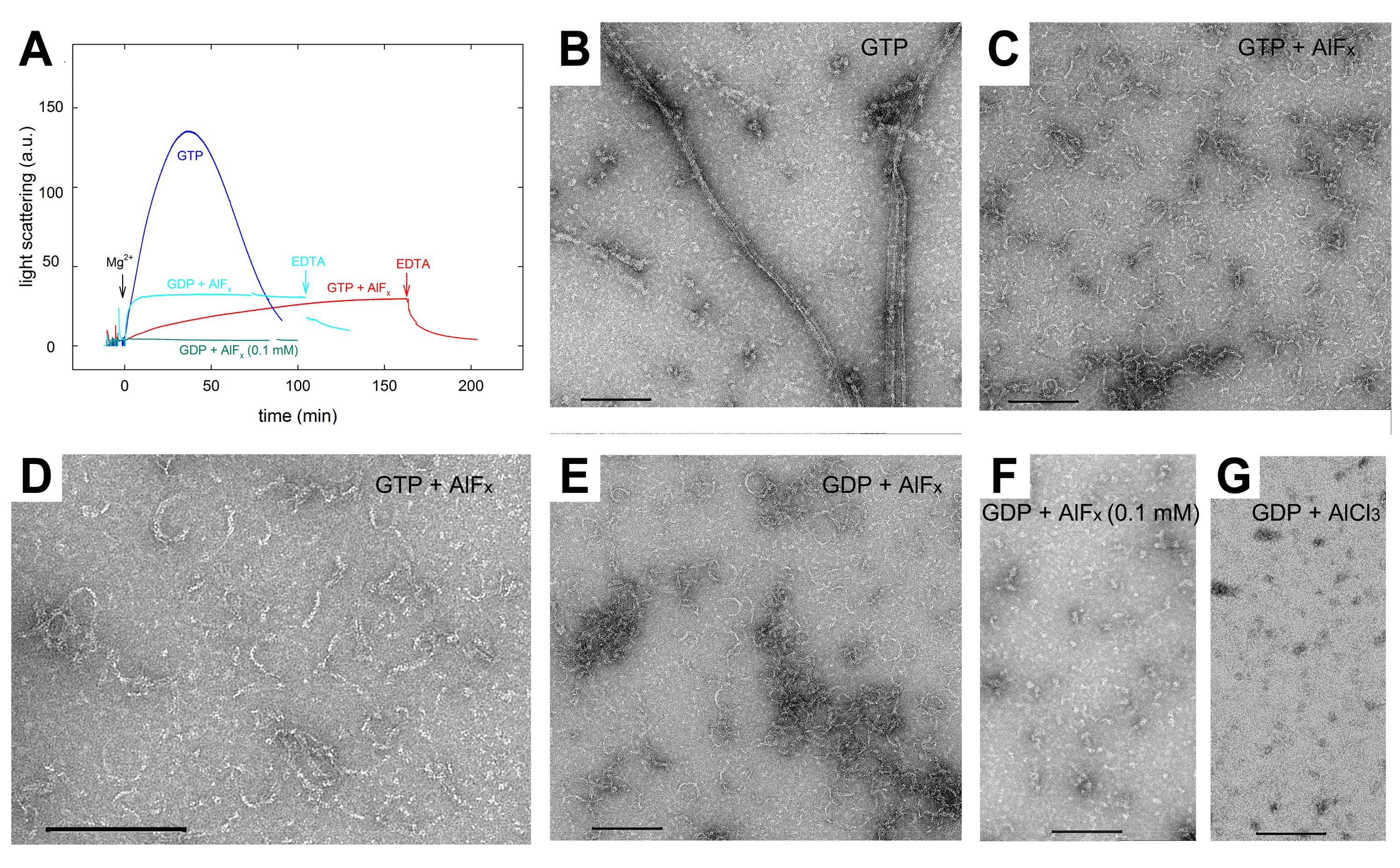

Supplement: S2 Fig — (A) Light scattering was employed to monitor the formation of polymers by the SaFtsZ core (50 μM) with 1 mM GTP (blue line), with 1 mM GTP plus 1 mM AlFx (red line), with 1 mM GDP plus 1 mM AlFx (magenta line) and with 1 mM GDP plus 0.1 mM AlFx (green line). Assembly was triggered by addition of 10 mM MgCl2 at time 0. Samples stabilized with 1 mM AlFx could be disassembled with 20 mM EDTA divalent metal chelator (indicated by the arrows). Numerical data for each curve can be found in S6 Data. The effects of adding AlFx to polymers preassembled with GTP could not be determined due to precipitate formation. (B) Electron micrograph of polymers formed with 1 mM GTP. (C) Polymers formed with 1 mM GTP + 1 mM AlFx. (D) Enlarged view with 1 mM GTP + 1 mM AlFx. (E) Polymers formed with 1 mM GDP + 1 mM AlFx. (F) Polymers formed with 1 mM GDP + 0.1 mM AlFx. (G) Polymers formed with 1 mM GDP + 1 mM AlCl3 control. The bars indicate 200 nm. (TIF) [file pbio.3001497.s002.tif]

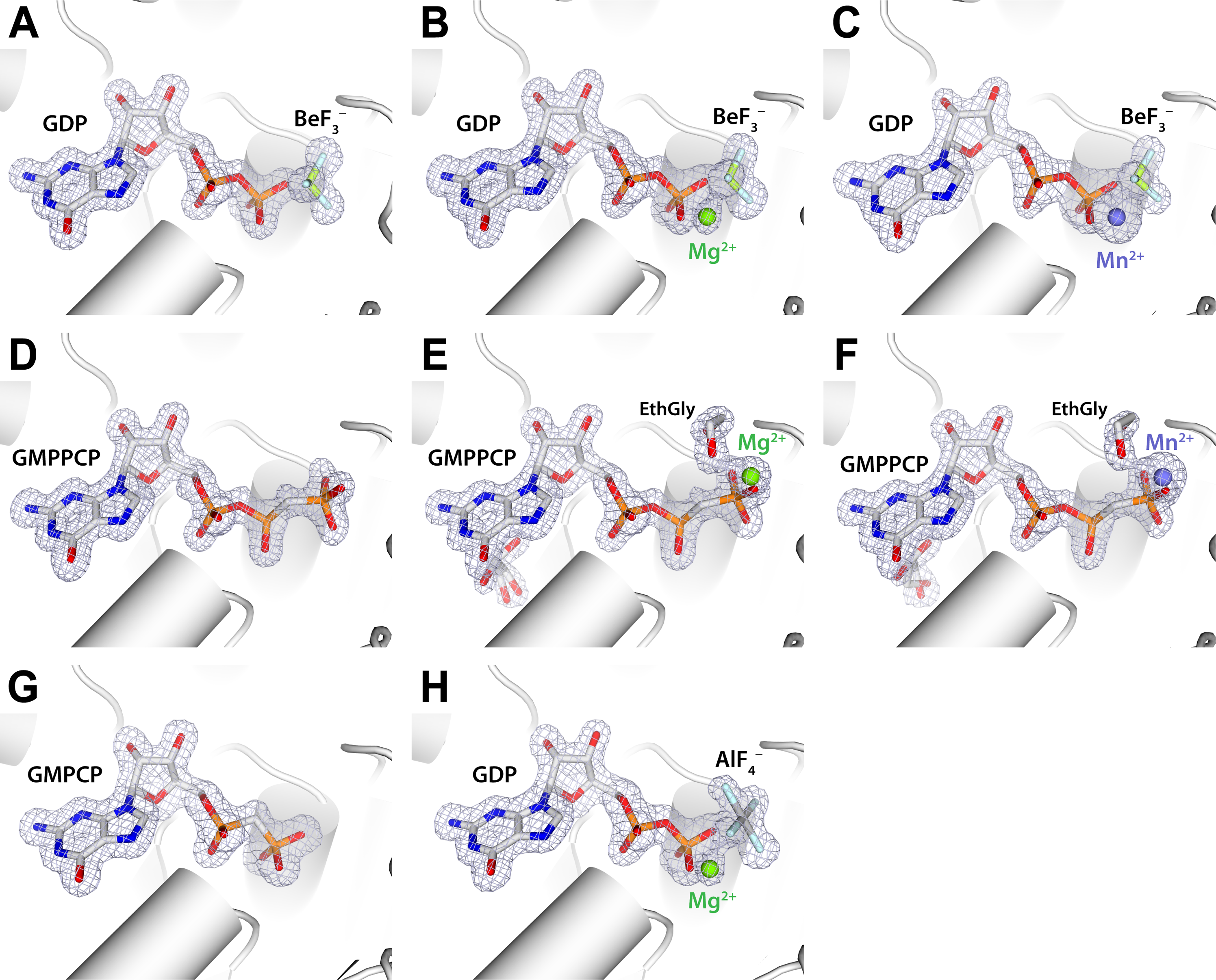

Supplement: S3 Fig — Expanded view of the nucleotide binding pocket, with polder OMIT electron density maps (blue mesh) contoured at 3 sigma around the different GTP mimetics and ions. (TIF) [file pbio.3001497.s003.tif]

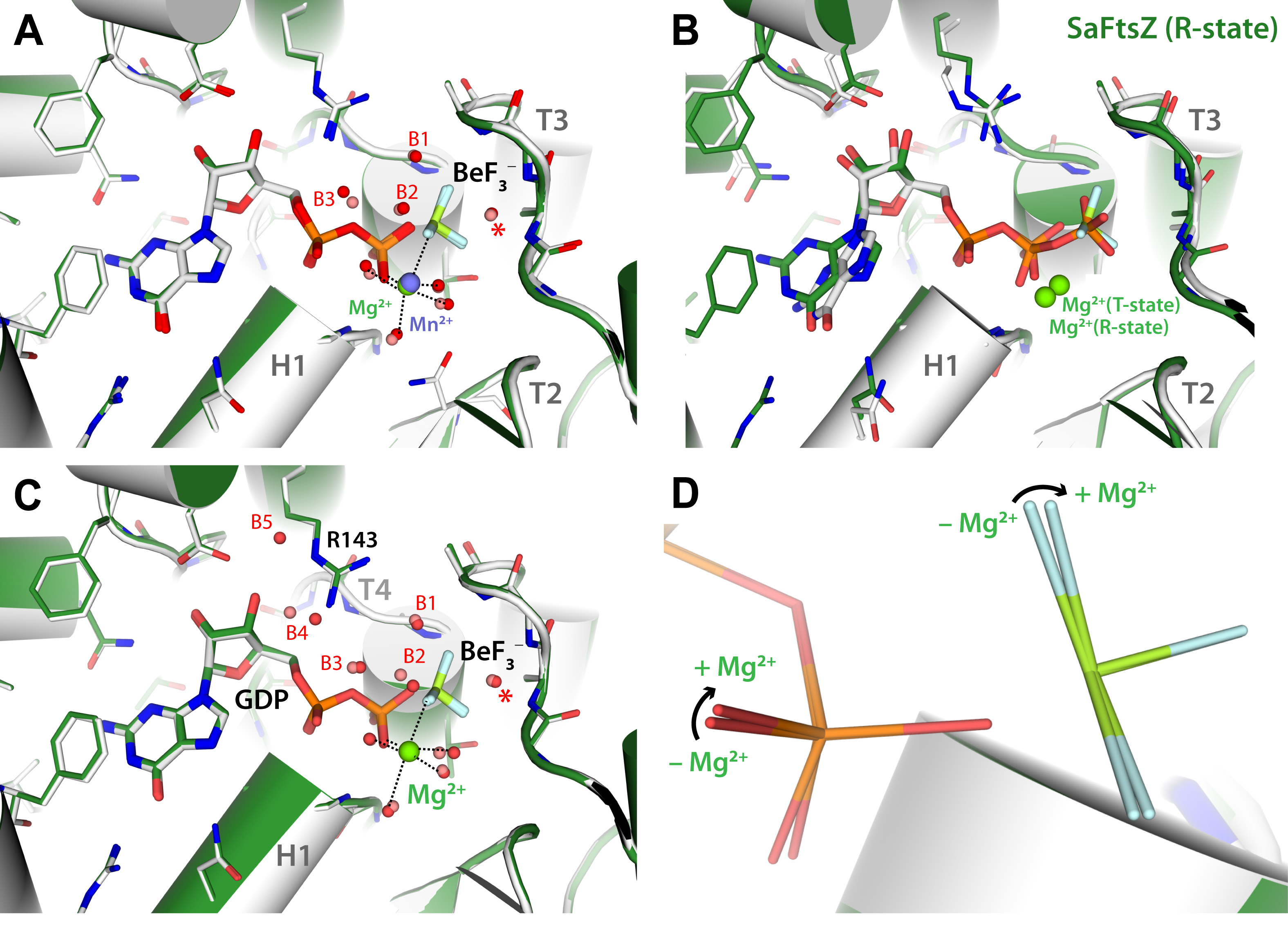

Supplement: S4 Fig — In all panels, the SaFtsZ complexed with GDP, BeF3−, and Mg2+ is shown in gray, and water molecules in this structure appear as red spheres. (A) Superposition onto the filament structure of SaFtsZ complexed with GDP, BeF3−, and Mn2+ (dark green for protein, salmon spheres for water). (B) Superposition onto the structure of SaFtsZ complexed with GTP and Mg2+ in the R conformation (PDB 5MN7, dark green). (C) Superposition onto the filament structure of SaFtsZ complexed with GDP and BeF3− in the absence of Mg2+ (dark green for protein, salmon spheres for water). (D) Close-up view of panel C superposition around BeF3− and the β-phosphate. (TIF) [file pbio.3001497.s004.tif]

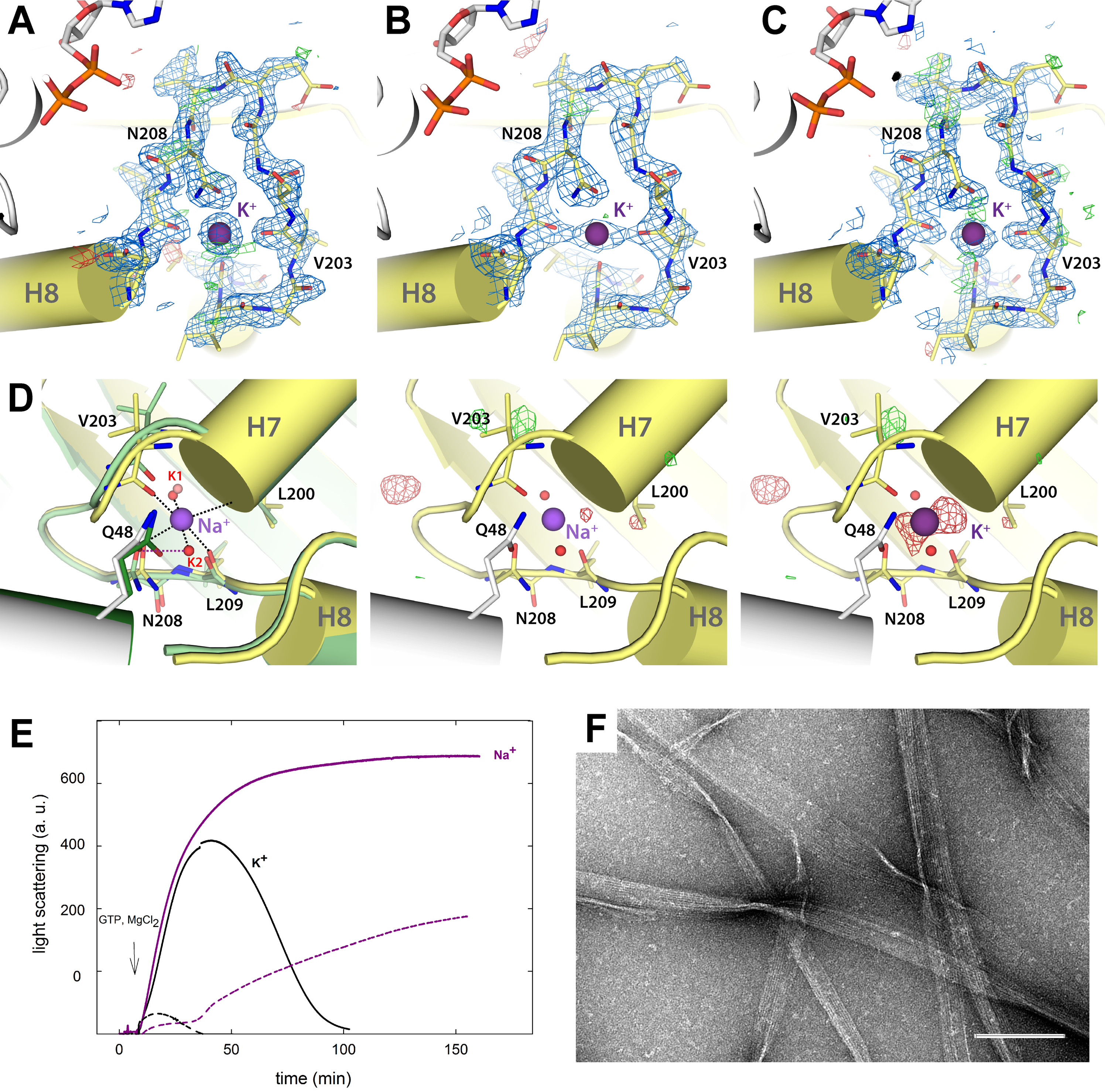

Supplement: S5 Fig — (A) SaFtsZ structure in complex with GDP, crystallized in the presence of 1 mM EDTA and 50 mM KCl. (B) SaFtsZ structure in complex with GDP of crystals soaked in 10 mM EGTA and 50 mM KCl. (C) SaFtsZ structure in complex with GDP of crystals soaked in 10 mM CyDTA and 50 mM KCl. (D) SaFtsZ structure in complex with GDP crystallized in the presence of 150 mM NaCl refined with Na+ (left and middle panels) or K+ (right panel) in the T7 loop. In all cases, 2Fo-Fc (blue) and Fo-Fc (green for positive, red for negative values) maps are contoured at 1.5 and 3.0 sigma, respectively. In the left panel, the structure (yellow and gray) is superposed to that in complex with GDP, BeF3−, and Mg2+. (E) Light scattering assembly time courses of SaFtsZ (50 μM) in K+ (black lines) and Na+-containing (pink lines) MES assembly buffers at 25°C. GTP (1 mM) was added, and assembly was triggered by addition of 10 mM MgCl2 (solid lines) or 5 mM MgCl2 (dashed lines) as indicated by the arrow. Numerical data for each curve can be found in S7 Data. (F) Representative electron micrograph of SaFtsZ in Na+ buffer with 10 mM MgCl2, to be compared with polymers formed in K+ buffer (Fig 1C). The bar indicates 200 nm. Notice the similarity of these scattering and electron microscopy results of SaFtsZ in Na+ buffer with those of the Q48A and R143K mutants in K+ buffer (Figs 6 and S8). (TIF) [file pbio.3001497.s005.tif]

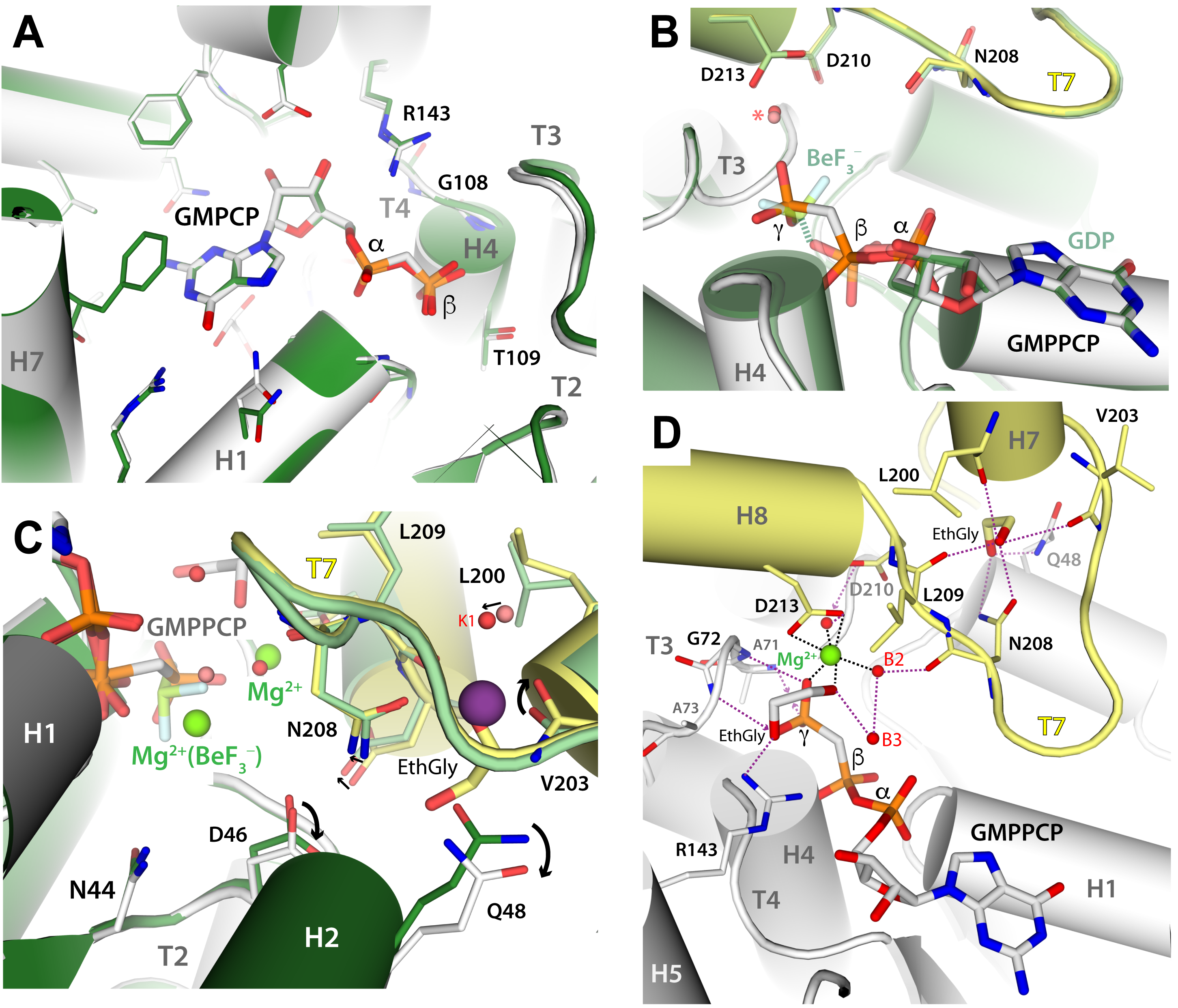

Supplement: S6 Fig — Bottom and top monomers are gray and yellow, while dark and light green is used for bottom and top monomers of structures complexed to BeF3−. Water molecules are red, while salmon is used for solvent molecules in structures complexed to BeF3−. Mg2+ and K+ appear as green and purple spheres, respectively. (A) Structure complexed to GMPCP superimposed onto that complexed with GDP, both in the absence of Mg2+. (B) Structure complexed to GMPPCP superimposed onto that in complex with GDP and BeF3−, both in the absence of Mg2+. (C) Structure complexed to GMPPCP superimposed onto that in complex with GDP and BeF3−, both in the presence of Mg2+. (D) Close-up view of the structure complexed to GMPPCP and Mg2+. Coordination contacts and H-bonds are shown as black and purple dash lines, respectively. (TIF) [file pbio.3001497.s006.tif]

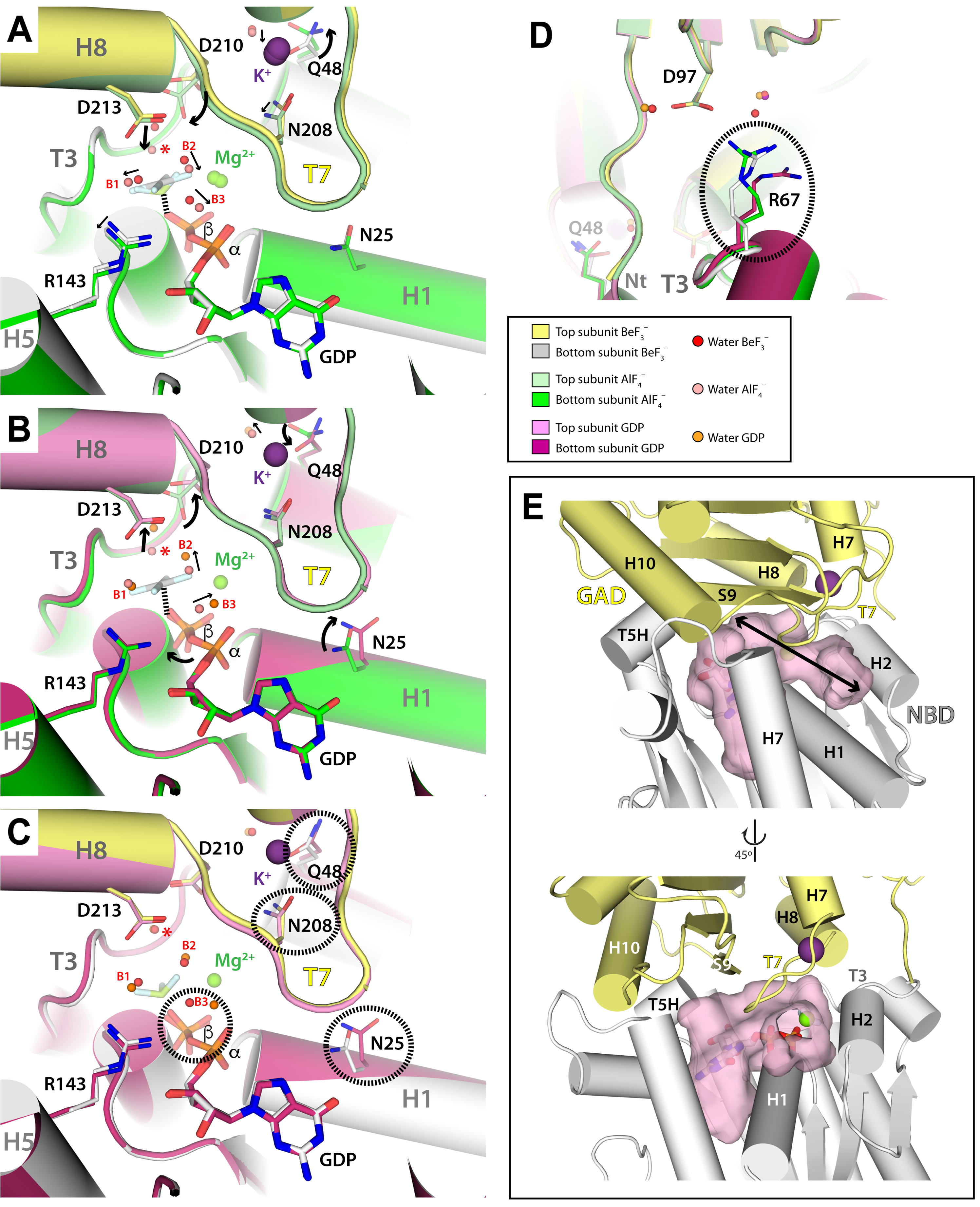

Supplement: S7 Fig — (A) Superposition of the SaFtsZ structure in complex with GDP, BeF3−, and Mg2+ (gray and yellow for bottom and top monomers, red spheres for water) onto that in complex with GDP, AlF4−, and Mg2+ (green and light green for bottom and top monomers, salmon spheres for water). Black arrows indicate conformational rearrangements between both structures. (B) Superposition of the SaFtsZ structure in complex with GDP, AlF4−, and Mg2+ (green and light green for bottom and top monomers, salmon spheres for water) onto that in complex with GDP alone (purple and pink for bottom and top monomers, violet spheres for water). (C) Superposition of the SaFtsZ structure in complex with GDP, BeF3−, and Mg2+ (gray and yellow for bottom and top monomers, red spheres for water) onto that in complex with GDP alone (purple and pink for bottom and top monomers, violet spheres for water). (D) Superposition of the 3 structures in panels A–C around residue R67. (E) Nucleotide-binding pocket (pink) formed at the interface between adjacent monomers, showing a pore from the solvent into the Mg2+ binding site. T5H is a mini-helix within loop T5. (TIF) [file pbio.3001497.s007.tif]

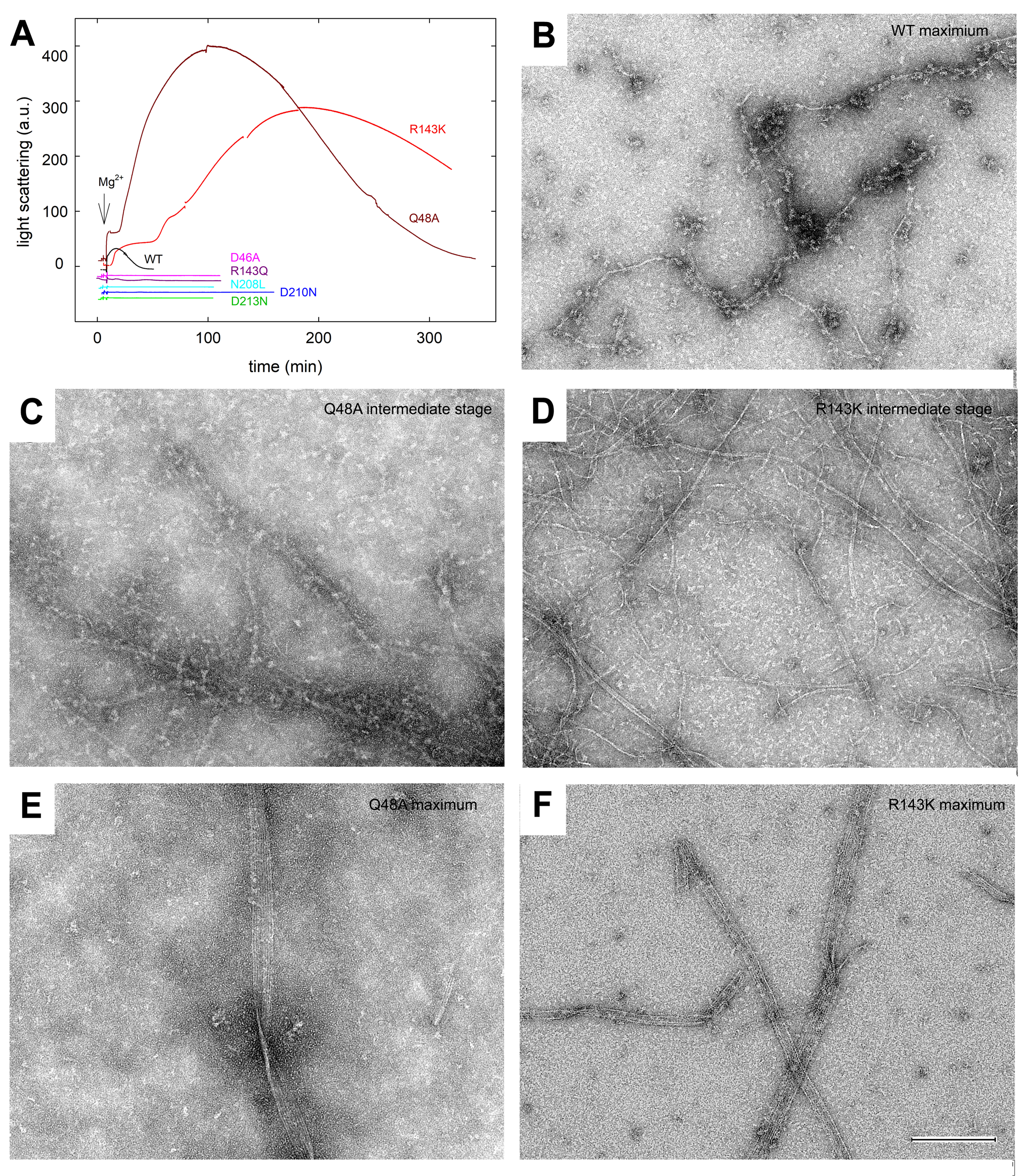

Supplement: S8 Fig — (A) Light scattering time courses during assembly of wild-type protein (black trace, 50 μM), D46A, Q48A, R143Q, R143K, N201A, D210N, and D213N (color traces, 50 μM each) in MES assembly buffer at 25°C, to which 2 mM GTP was added; assembly was triggered by addition of 5 mM MgCl2 at time 0. Numerical data for each curve can be found in S8 Data. (B) Negatively stained electron micrograph of wild-type SaFtsZ polymers, at maximum light scattering. (C) Mutant Q48A at the intermediate scattering plateau. (D) R143K at the intermediate scattering plateau. (E) Q48A at maximum scattering. (F) R143A at maximum scattering. The bar indicates 200 nm. (TIF) [file pbio.3001497.s008.tif]

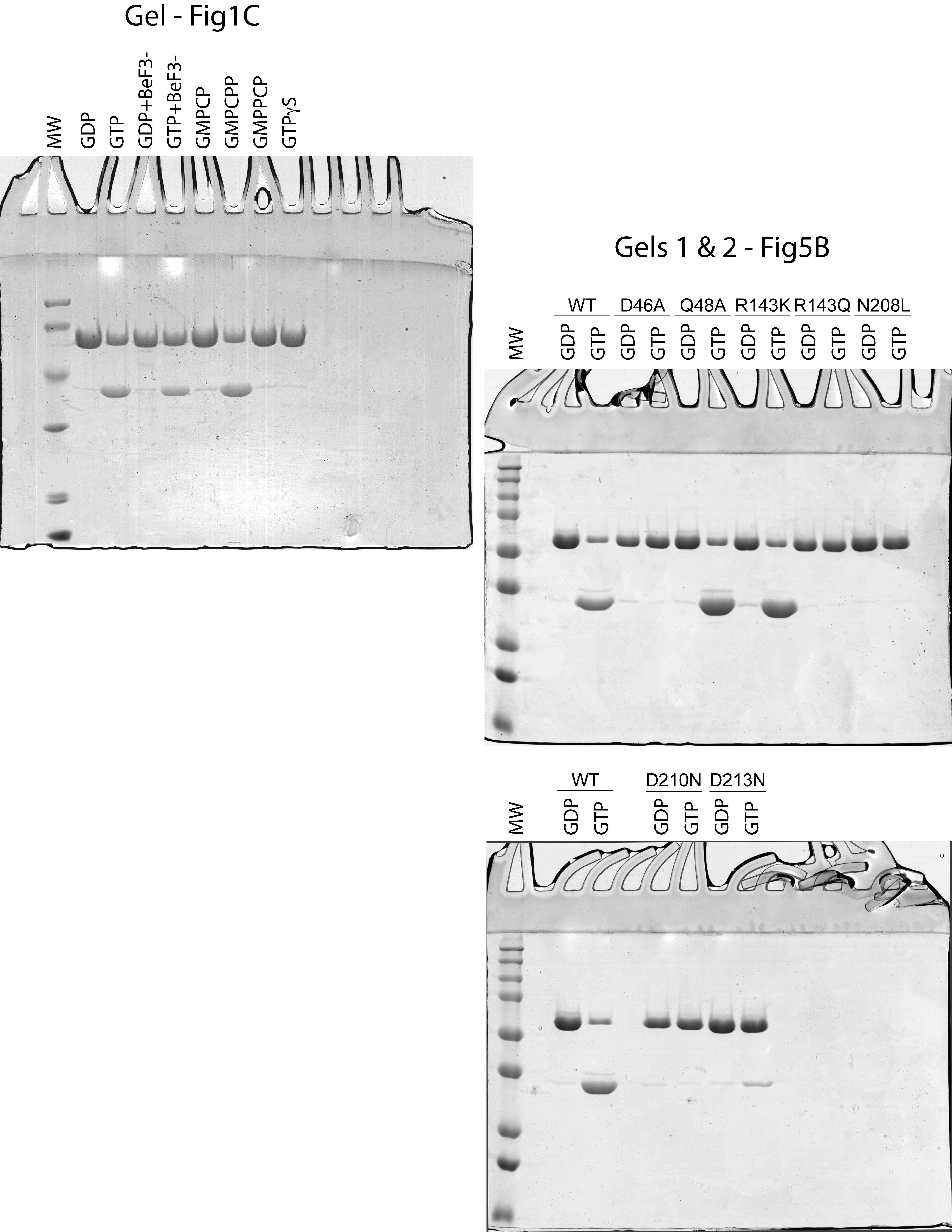

Supplement: S1 Raw images — Gel Fig 1C: Sedimentation of polymers formed by SaFtsZ (31 μM) with different guanine nucleotides and mimetics (1 mM each, except 0.1 mM GMPCP and GMPCPP); pellet and supernatant samples were consecutively loaded with a 15-min time shift in SDS-PAGE. MW corresponds to molecular weight markers. Gels 1 and 2 Fig 5B: Sedimentation assays of polymer formation by wild-type and mutant SaFtsZ (31 μM) with 10 mM MgCl2 by high-speed centrifugation at the time of maximal scattering. For each sample, the left and right lanes contain 1 mM GDP and GTP, respectively; pellet and supernatant samples were consecutively loaded with a 15-min time shift in SDS-PAGE. MW corresponds to molecular weight markers. GMPCP, guanosine-5′-[(α,β)-methyleno]diphosphate; GMPCPP, guanosine-5′-[(α,β)-methyleno]triphosphate; SaFtsZ, Staphylococcus aureus FtsZ core (residues 12 to 316). (TIF) [file pbio.3001497.s027.tif]
