# Supplementary material for: FtsZ filament structures in different nucleotide states reveal the mechanism of assembly dynamics
Source: PLoS Biol. 2022 Mar 21;20(3):e3001497. doi: 10.1371/journal.pbio.3001497 (PMC8936486; doi:10.1371/journal.pbio.3001497)
Supplement: S1 Table — (DOCX) [file pbio.3001497.s009.docx]

## S1 Table. Data collection and refinement statistics (Part I)

|  | | **FtsZ_GDP**  **BeF_3_^-^** | FtsZ_GDP  BeF_3_^-^_Mg^2+^ | FtsZ_GDP  BeF_3_^-^_Mn^2+^ | FtsZ_GDP  AlF_4_^-^_Mg^2+^ |
| --- | --- | --- | --- | --- | --- |
| **Wavelength** | | 0.9792 | 0.9724 | 0.9792 | 0.9787 |
| **Resolution range** | | 40.99 - 1.45  (1.502 - 1.45) | 41.18 - 1.75  (1.813 - 1.75) | 41.14 - 1.75  (1.81 - 1.75) | 40.09 - 1.62 (1.68 - 1.62) |
| **Space group** | | C2 | C2 | C2 | C2 |
| **Unit cell** | **a, b, c (Å) α, β, γ (º)** | 71.7 51.1 87.8 90 111.0 90 | 70.6 52.3 87.7 90 110.0 90 | 72.0 51.5 88.0 90 110.8 90 | 70.1 52.0 87.5  90 109.8 90 |
| **Total reflections** | | 202804 (20805) | 58891 (5604) | 140375 (13564) | 125039 (11208) |
| **Unique reflections** | | 51784 (5181) | 29850 (2892) | 30098 (2991) | 37431 (3590) |
| **Multiplicity** | | 3.9 (4.0) | 2.0 (1.9) | 4.7 (4.5) | 3.3 (3.1) |
| **Completeness (%)** | | 98.13 (98.66) | 98.11 (96.69) | 98.37 (99.17) | 99.24 (95.65) |
| **Mean I/sigma(I)** | | 12.83 (0.64) | 10.02 (1.73) | 8.15 (0.69) | 11.90 (1.86) |
| **Wilson B-factor** | | 25.89 | 23.25 | 34.19 | 22.43 |
| **R-merge** | | 0.050 (1.759) | 0.040 (0.395) | 0.096 (1.439) | 0.060 (0.676) |
| **R-meas** | | 0.059 (2.031) | 0.057 (0.559) | 0.108 (1.625) | 0.072 (0.814) |
| **R-pim** | | 0.0298 (1) | 0.040 (0.395) | 0.048 (0.738) | 0.039 (0.449) |
| **CC1/2** | | 0.998 (0.306) | 0.997 (0.786) | 0.997 (0.334) | 0.996 (0.797) |
| **Reflections in refinement** | | 51772 (5168) | 29842 (2890) | 30097 (2991) | 37413 (3583) |
| **Reflections for R-free** | | 2585 (258) | 1078 (105) | 1504 (149) | 1795 (198) |
| **R-work** | | 0.17 (0.67) | 0.16 (0.25) | 0.16 (0.32) | 0.16 (0.33) |
| **R-free** | | 0.20 (0.62) | 0.20 (0.28) | 0.21 (0.36) | 0.20 (0.37) |
| **Number of atoms** | | 2490 | 2497 | 2357 | 2536 |
| **macromolecules** | | 2235 | 2239 | 2206 | 2256 |
| **ligands** | | 33 | 34 | 34 | 35 |
| **solvent** | | 222 | 224 | 117 | 245 |
| **Protein residues** | | 307 | 308 | 305 | 307 |
| **RMS(bonds)** | | 0.009 | 0.009 | 0.030 | 0.010 |
| **RMS(angles)** | | 1.18 | 1.17 | 3.24 | 1.10 |
| **Ramachandran favored (%)** | | 99.02 | 96.69 | 98.02 | 99.34 |
| **Ramachandran allowed (%)** | | 0.98 | 1.31 | 1.98 | 0.66 |
| **Ramachandran outliers (%)** | | 0.00 | 0.00 | 0.00 | 0.00 |
| **Rotamer outliers (%)** | | 0.42 | 1.69 | 0.00 | 0.84 |
| **Clashscore** | | 2.86 | 3.74 | 6.68 | 2.83 |
| **Average B-factor** | | 31.73 | 31.77 | 43.38 | 30.09 |
| **macromolecules** | | 31.30 | 31.24 | 43.50 | 29.45 |
| **ligands** | | 19.67 | 19.60 | 30.35 | 18.86 |
| **solvent** | | 37.84 | 38.93 | 44.76 | 37.51 |
| **PDB code** | | 7OHH | 7OHK | 7OHL | 7OHN |

## S1 Table (continued). Data collection and refinement statistics (Part II)

|  | | FtsZ_GMPPCP | FtsZ_GMPPCP  Mg^2+^ | FtsZ_GMPPCP  Mn^2+^ | FtsZ_GMPCP |
| --- | --- | --- | --- | --- | --- |
| **Wavelength** | | 0.9793 | 0.9792 | 0.9793 | 0.9792 |
| **Resolution range** | | 41.12 - 1.57  (1.62 - 1.57) | 41.22 - 1.52  (1.57 - 1.52) | 33.92 - 1.45  (1.50 - 1.45) | 41.13 - 1.65  (1.71 - 1.65) |
| **Space group** | | C2 | C2 | C2 | C2 |
| **Unit cell** | **a, b, c (Å) α, β, γ (º)** | 71.0 51.9 87.6  90 110.2 90 | 70.6 51.9 87.8  90 110.1 90 | 70. 9 52.0 87.9 90 110.3 90 | 70.8 52.2 86.0  90 109.2 90 |
| **Total reflections** | | 170437 (17336) | 204621 (19488) | 233424 (23457) | 117877 (11309) |
| **Unique reflections** | | 41443 (4104) | 45499 (4545) | 52628 (5227) | 34420 (3365) |
| **Multiplicity** | | 4.1 (4.2) | 4.5 (4.3) | 4.4 (4.5) | 3.4 (3.4) |
| **Completeness (%)** | | 98.82 (99.15) | 98.71 (98.57) | 98.68 (98.65) | 96.17 (94.68) |
| **Mean I/sigma(I)** | | 12.03 (1.52) | 14.62 (1.82) | 9.88 (1.11) | 14.44 (2.15) |
| **Wilson B-factor** | | 20.91 | 17.93 | 20.77 | 20.35 |
| **R-merge** | | 0.060 (0.690) | 0.056 (0.637) | 0.072 (1.27) | 0.0514 (0.616) |
| **R-meas** | | 0.069 (0.789) | 0.064 (0.726) | 0.081 (1.447) | 0.061 (0.735) |
| **R-pim** | | 0.033 (0.376) | 0.029 (0.341) | 0.037 (0.671) | 0.032 (0.396) |
| **CC1/2** | | 0.998 (0.793) | 0.999 (0.792) | 0.998 (0.620) | 0.998 (0.777) |
| **Reflections in refinement** | | 41411 (4104) | 45482 (4543) | 52564 (5200) | 34414 (3364) |
| **Reflections for R-free** | | 1998 (191) | 2152 (236) | 2572 (273) | 1734 (157) |
| **R-work** | | 0.14 (0.22) | 0.15 (0.25) | 0.16 (0.30) | 0.14 (0.22) |
| **R-free** | | 0.18 (0.28) | 0.17 (0.28) | 0.18 (0.33) | 0.17 (0.26) |
| **Number of atoms** | | 2539 | 2602 | 2518 | 2540 |
| **macromolecules** | | 2246 | 2266 | 2264 | 2267 |
| **ligands** | | 33 | 49 | 49 | 37 |
| **solvent** | | 260 | 287 | 205 | 236 |
| **Protein residues** | | 308 | 307 | 307 | 307 |
| **RMS(bonds)** | | 0.009 | 0.009 | 0.008 | 0.010 |
| **RMS(angles)** | | 1.13 | 1.17 | 1.01 | 1.13 |
| **Ramachandran favored (%)** | | 99.02 | 98.69 | 99.02 | 99.02 |
| **Ramachandran allowed (%)** | | 0.98 | 1.31 | 0.98 | 0.98 |
| **Ramachandran outliers (%)** | | 0.00 | 0.00 | 0.00 | 0.00 |
| **Rotamer outliers (%)** | | 0.84 | 0.00 | 0.42 | 0.41 |
| **Clashscore** | | 2.85 | 2.80 | 2.58 | 3.44 |
| **Average B-factor** | | 27.19 | 24.66 | 31.63 | 26.91 |
| **macromolecules** | | 26.12 | 23.63 | 31.18 | 26.31 |
| **ligands** | | 17.90 | 17.45 | 24.67 | 18.24 |
| **solvent** | | 37.61 | 34.01 | 38.29 | 34.01 |
| **PDB code** | | 7OMJ | 7OMP | 7OMQ | 7OJZ |

## S1 Table (continued). Data collection and refinement statistics (Part III)

|  | | FtsZ_GDP  (NaCl) | FtsZ_GDP  (10 mM CyDTA) | FtsZ_GDP  (10 mM EGTA) | FtsZ_GDP  (10 mM EDTA) |
| --- | --- | --- | --- | --- | --- |
| **Wavelength** | | 0.9792 | 0.9787 | 0.9787 | 0.9792 |
| **Resolution range** | | 41.61 - 1.90  (1.96 - 1.90) | 36.16 - 1.69  (1.75 - 1.69) | 40.58 - 2.32  (2.40 - 2.32) | 40.19 - 1.79  (1.85 - 1.79) |
| **Space group** | | C2 | C2 | C2 | C2 |
| **Unit cell** | **a, b, c (Å) α, β, γ (º)** | 69.2 53.7 85.3  90 108.2 90 | 72.4 50.2 88.0  90 111.4 90 | 71.8 50.9 88.5 90 111.0 90 | 71.2 51.6 87.9  90 110.6 90 |
| **Total reflections** | | 78877 (8012) | 102023 (9125) | 39160 (3349) | 95781 (8935) |
| **Unique reflections** | | 23465 (2315) | 31990 (2889) | 12638 (1114) | 27563 (2590) |
| **Multiplicity** | | 3.4 (3.5) | 3.2 (3.2) | 3.1 (3.0) | 3.5 (3.4) |
| **Completeness (%)** | | 99.32 (99.91) | 96.53 (88.44) | 96.46 (85.48) | 95.60 (91.42) |
| **Mean I/sigma(I)** | | 14.40 (1.72) | 13.23 (1.97) | 6.94 (1.77) | 11.33 (3.18) |
| **Wilson B-factor** | | 30.07 | 28.63 | 36.52 | 19.48 |
| **R-merge** | | 0.056 (0.653) | 0.044 (0.460) | 0.128 (0.751) | 0.070 (0.278) |
| **R-meas** | | 0.067 (0.773) | 0.053 (0.556) | 0.156 (0.918) | 0.082 (0.329) |
| **R-pim** | | 0.036 (0.410) | 0.029 (0.308) | 0.087 (0.520) | 0.043 (0.174) |
| **CC1/2** | | 0.999 (0.759) | 0.998 (0.882) | 0.986 (0.648) | 0.996 (0.951) |
| **Reflections in refinement** | | 23456 (2315) | 31973 (2884) | 12599 (1101) | 27140 (2590) |
| **Reflections for R-free** | | 1203 (130) | 1549 (152) | 611 (56) | 1406 (131) |
| **R-work** | | 0.17 (0.29) | 0.20 (0.26) | 0.23 (0.36) | 0.15 (0.22) |
| **R-free** | | 0.23 (0.37) | 0.24 (0.29) | 0.28 (0.36) | 0.20 (0.27) |
| **Number of atoms** | | 2490 | 2420 | 2338 | 2549 |
| **macromolecules** | | 2246 | 2225 | 2211 | 2240 |
| **ligands** | | 37 | 33 | 33 | 37 |
| **solvent** | | 207 | 162 | 94 | 272 |
| **Protein residues** | | 307 | 307 | 306 | 308 |
| **RMS(bonds)** | | 0.007 | 0.011 | 0.001 | 0.010 |
| **RMS(angles)** | | 0.95 | 1.22 | 0.43 | 1.14 |
| **Ramachandran favored (%)** | | 99.34 | 99.34 | 98.68 | 98.69 |
| **Ramachandran allowed (%)** | | 0.66 | 0.66 | 1.32 | 1.31 |
| **Ramachandran outliers (%)** | | 0.00 | 0.00 | 0.00 | 0.00 |
| **Rotamer outliers (%)** | | 0.84 | 0.85 | 0.00 | 0.42 |
| **Clashscore** | | 6.76 | 6.41 | 10.23 | 4.60 |
| **Average B-factor** | | 33.76 | 34.75 | 40.16 | 25.97 |
| **macromolecules** | | 33.44 | 34.64 | 40.28 | 25.27 |
| **ligands** | | 22.68 | 26.20 | 34.05 | 15.73 |
| **solvent** | | 39.15 | 37.96 | 39.48 | 33.17 |
| **PDB code** | | 7OI2 | 7ON2 | 7ON3 | 7ON4 |

**S1 Table (continued). Data collection and refinement statistics (Part IV)**

|  | | FtsZ(D210N)_GDP | FtsZ(R143K)_GDP | FtsZ(Q48A)_GDP | FtsZ(D46A)_GDP |
| --- | --- | --- | --- | --- | --- |
| **Wavelength** | | 0.9792 | 0.9792 | 0.9792 | 0.9792 |
| **Resolution range** | | 40.46 - 2.22  (2.30 - 2.22) | 41.70 - 1.70  (1.76 - 1.70) | 39.99 - 1.95  (2.02 - 1.95) | 41.5 - 1.82  (1.885 - 1.82) |
| **Space group** | | C2 | C2 | C2 | C2 |
| **Unit cell** | **a, b, c (Å) α, β, γ (º)** | 67.0 53.2 84.1  90 105.7 90 | 69.0 54.0 85.1  90 108.1 90 | 71.8 50.9 88.2  90 111.0 90 | 68.1 53.9 84.8  90 107.2 90 |
| **Total reflections** | | 47618 (4673) | 107667 (9606) | 69426 (6752) | 88222 (8750) |
| **Unique reflections** | | 14162 (1375) | 32698 (3217) | 21687 (2151) | 26341 (2569) |
| **Multiplicity** | | 3.4 (3.4) | 3.3 (3.0) | 3.2 (3.1) | 3.3 (3.4) |
| **Completeness (%)** | | 99.52 (99.42) | 99.31 (98.17) | 98.55 (97.99) | 97.83 (96.98) |
| **Mean I/sigma(I)** | | 9.47 (1.77) | 12.53 (1.82) | 9.19 (1.78) | 12.85 (1.44) |
| **Wilson B-factor** | | 38.76 | 25.02 | 28.73 | 31.71 |
| **R-merge** | | 0.080 (0.714) | 0.053 (0.505) | 0.0781 (0.491) | 0.0635 (0.728) |
| **R-meas** | | 0.095 (0.848) | 0.064 (0.617) | 0.093 (0.590) | 0.075 (0.865) |
| **R-pim** | | 0.051 (0.452) | 0.034 (0.350) | 0.051 (0.324) | 0.040 (0.463) |
| **CC1/2** | | 0.996 (0.704) | 0.998 (0.814) | 0.994 (0.782) | 0.997 (0.840) |
| **Reflections in refinement** | | 14158 (1375) | 32689 (3213) | 21586 (2149) | 25916 (2569) |
| **Reflections for R-free** | | 715 (67) | 1661 (145) | 1111 (102) | 1334 (129) |
| **R-work** | | 0.18 (0.26) | 0.17 (0.36) | 0.17 (0.29) | 0.18 (0.33) |
| **R-free** | | 0.24 (0.34) | 0.21 (0.41) | 0.217(0.35) | 0.23 (0.40) |
| **Number of atoms** | | 2366 | 2553 | 2471 | 2408 |
| **macromolecules** | | 2231 | 2234 | 2235 | 2222 |
| **ligands** | | 46 | 37 | 37 | 37 |
| **solvent** | | 89 | 282 | 199 | 149 |
| **Protein residues** | | 307 | 307 | 308 | 306 |
| **RMS(bonds)** | | 0.003 | 0.007 | 0.008 | 0.011 |
| **RMS(angles)** | | 0.61 | 0.94 | 0.90 | 1.15 |
| **Ramachandran  favored (%)** | | 97.70 | 99.02 | 99.02 | 99.01 |
| **Ramachandran  allowed (%)** | | 2.30 | 0.98 | 0.98 | 0.99 |
| **Ramachandran  outliers (%)** | | 0.00 | 0.00 | 0.00 | 0.00 |
| **Rotamer outliers (%)** | | 0.00 | 1.27 | 0.00 | 0.85 |
| **Clashscore** | | 3.29 | 1.97 | 3.73 | 3.75 |
| **Average B-factor** | | 46.82 | 30.72 | 31.75 | 44.06 |
| **macromolecules** | | 46.66 | 29.88 | 31.60 | 44.13 |
| **ligands** | | 55.77 | 20.74 | 22.74 | 35.46 |
| **solvent** | | 46.40 | 38.64 | 35.14 | 45.11 |
| **PDB code** | | 7OJA | 7OJB | 7OJC | 7OJD |
