# Supplementary material for: FtsZ filament structures in different nucleotide states reveal the mechanism of assembly dynamics
Source: PLoS Biol. 2022 Mar 21;20(3):e3001497. doi: 10.1371/journal.pbio.3001497 (PMC8936486; doi:10.1371/journal.pbio.3001497)
Supplement: S2 Table — (PDF) [file pbio.3001497.s010.pdf]

S2 Table. Water molecules at the subunit interface

FtsZ_GDP + BeF_3^{-}+ Mg^{2+}			FtsZ_GDP + AlF_4^{-} + Mg^{2+}			FtsZ_GDP		
7OHK			7OHN			6RVN		
NBD[1]	W[2,3]	GAD[1]	NBD[1]	W[2,3]	GAD[1]	NBD[1]	W[2,3]	GAD[1]
N25(OD1)	602	G205(O)<br>G206(O)	N25(OD1)	563	G205(O)<br>G206(O)	N25(OD1)	506	G205(O)<br>G206(O)
Mg^{2+}	630<br>[M2]	D46(OD2)<br>N44(ND2)	Mg^{2+}	628<br>[M2]	N44(ND2)	----	517	----
Mg^{2+}<br>D46(OD2)<br>BEF(F3)	626<br>[M1]	D210(OD2)	Mg^{2+}<br>D46(OD2)<br>ALF(F1)	516<br>[M1]	D210(OD2)	D46(OD2)	643	D210(OD2)
BEF(F3)<br>GDP(O2A1)<br>GDP(O2B1)<br>Mg^{2+}	582<br>[M4]	N208(O)	ALF(F1)<br>GDP(O2A1)<br>GDP(O2B1)<br>Mg^{2+}	556<br>[M4]	N208(O)	GDP(O2A1)<br>GDP(O2B1)	604	N208(O)
BEF(F2)	545<br>[B1]	D213(OD1)	ALF(F3)	574<br>[B1]	D213(OD1)	----	507<br>[B1]	D213(OD1)
BEF(F3)	566<br>[B2]	N208(O)<br>D213(OD2)	ALF(F1)<br>ALF(F3)	585<br>[B2]	N208(O)<br>D213(OD2)	----	582<br>[B2]	N208(O)<br>D213(OD2)
BEF(F3)<br>G72(N)	617<br>[*]	D210(OD1)<br>D213(OD1)	ALF(F1)<br>G72(N)	578<br>[*]	D210(OD1)<br>D213(OD1)	G72(N)	594	D210(OD1)<br>D213(OD1)
R29(NH1)<br>N25(ND2)<br>GDP(O6)	610	E206(O)	R29(NH1)<br>N25(ND2)<br>GDP(O6)	566	E206(O)	R29(NH1)<br>N25(ND2)<br>GDP(O6)	544	E206(O)
E139(OE1)<br>R143(NE)	537<br>[B5]	F294(N)<br>M292(O)	E139(OE1)<br>R143(NE)	561<br>[B5]	F294(N)<br>M292(O)	E139(OE1)<br>R143(NE)	562<br>[B5]	F294(N)<br>M292(O)
R143(NH1)	664	N291(OD1)	R143(NH1)	656	N291(OD1)	R143(NH1)	659	N291(OD1)
R143(NH2)	571<br>[B4]	F294(O)	R143(NH2)	536<br>[B4]	F294(O)	R143(NH2)	568<br>[B4]	F294(O)
R143(N)<br>E139(O)	547	V290(O)	R143(N)<br>E139(O)	592	V290(O)	R143(N)<br>E139(O)	532	V290(O)
D46(OD1)	526	D210 (OD2)	D46(OD1)	613	D210 (OD2)	D46(OD1)	519	D210 (OD2)
G68(O)	521	H10(N)	G68(O)	558	H10(N)	G68(O)	537	H10(N)

[1]Residue followed by atom, the latter in parenthesis.

[2]Labels used in Figures are in brackets.

[3]Additional waters in 7OHK are M3:619, K1:661, K2:604, B3:665; in 7OHN are M3:631, K1:658, K2:681, B3:685.
