# Supplementary material for: FtsZ filament structures in different nucleotide states reveal the mechanism of assembly dynamics
Source: PLoS Biol. 2022 Mar 21;20(3):e3001497. doi: 10.1371/journal.pbio.3001497 (PMC8936486; doi:10.1371/journal.pbio.3001497)
Supplement: S3 Table — (PDF) [file pbio.3001497.s011.pdf]

S3 Table. GTPase activity of SaFtsZ and mutants under assembly conditions (min^{-1})[a]

Wild-type in K^{+} buffer	0.60 ± 0.03
Wild-type in Na^{+} buffer	0.05 ± 0.01
D46A	NS[b]
Q48A	0.08 ± 0.03
R143K	0.10 ± 0.02
D210N	NS[b]

[a]GTPase rate values are average and standard error from three independent experiments

[b]Non-Significant phosphate release after 3 hours
