# Supplementary material for: FtsZ filament structures in different nucleotide states reveal the mechanism of assembly dynamics
Source: PLoS Biol. 2022 Mar 21;20(3):e3001497. doi: 10.1371/journal.pbio.3001497 (PMC8936486; doi:10.1371/journal.pbio.3001497)
Supplement: S4 Table — (PDF) [file pbio.3001497.s012.pdf]

S4 Table. Primers used in this study

Primer name	Sequence
SaFtsZ-D46A-Fw	ATCGCTATCAACACA**GCC**GGTCAAGCTTTAAAC
SaFtsZ-D46A-Rv	GTTTAAAGCTTGACC**GGC**TGTGTTGATAGCGAT
SaFtsZ-Q48A-Fw	ATCAACACAGACGGT**GCA**GCTTTAAACTTATCT
SaFtsZ-Q48A-Rv	AGATAAGTTTAAAGC**TGC**ACCGTCTGTGTTGAT
SaFtsZ-R143K-Fw	TTTGAAGGACGTAAA**AAA**CAAACTCAAGCTGCT
SaFtsZ-R143K-Rv	AGCAGCTTGAGTTTG**TTT**TTTACGTCCTTCAAA
SaFtsZ-R143Q-Fw	TTTGAAGGACGTAAA**CAA**CAAACTCAAGCTGCT
SaFtsZ-R143Q-Rv	AGCAGCTTGAGTTTG**TTG**TTTACGTCCTTCAAA
SaFtsZ-N208L-Fw	GCTGTTTCTGGTGAAGTA**CTA**TTAGACTTTGCAGACGTT
SaFtsZ-N208L-Rv	AACGTCTGCAAAGTCTAA**AAG**TACTTCACCAGAAACAGC
SaFtsZ-D210N-Fw	CTGGTGAAGTAAACTTA**AAC**TTTGCAGACGTTAAGACAA
SaFtsZ-D210N-Rv	TTGTCTTAACGTCTGCAAA**GTT**TAAGTTTACTTCACCAG
SaFtsZ-D213N-Fw	GAAGTAAACTTAGACTTTGCA**AAC**GTTAAGACAATTATGTCTAAC
SaFtsZ-D213N-Rv	GTTAGACATAATTGTCTTAAC**GTT**TGCAAAGTCTAAGTTTACTTC
